# Biofilm formation of *Lactiplantibacillus plantarum* food isolates under flow and resistance to disinfectant agents

**DOI:** 10.1016/j.heliyon.2024.e38502

**Published:** 2024-09-26

**Authors:** P. Rashtchi, E. van der Linden, M. Habibi, T. Abee

**Affiliations:** a Physics and Physical Chemistry of Foods, Wageningen University, Wageningen, 6708WG, the Netherlands; b Food Microbiology, Wageningen University, Wageningen, 6708WG, the Netherlands

## Abstract

Bacterial biofilms formed in food processing environments can be resilient against cleaning and disinfection causing recontamination and spoilage of foods. We investigated the biofilm formation of six *Lactiplantibacillus plantarum* food spoilage isolates (FBR1-FBR6) using WCFS1 as a reference strain, and examined the impact of benzalkonium chloride (BKC) and peracetic acid (PAA) on planktonic and biofilm cells formed under static and dynamic flow conditions. We used a custom-designed setup composed of a 48-well plate with 0.8 ml culture volumes. We quantified biofilm formation under static and dynamic flow conditions with a flow rate of 3.2 ml/h using plate counting, Crystal Violet (CV) staining, and fluorescence staining techniques. Our findings revealed significant differences in biofilm formation and disinfectant resistance among studied strains and cell types. We observed that flow promoted biofilm formation in some strains and increased the number of culturable cells within biofilms in all strains. Furthermore, biofilm cells demonstrated higher resistance to disinfectants in comparison to planktonic cells for certain strains. Interestingly, cells from dispersed under flow biofilms show higher resistance to disinfectants than cells from static biofilms. The results indicate the importance of flow conditions in influencing *L. plantarum* food isolates biofilm formation and disinfection resistance, which may have implications for product contamination and spoilage risks.

## 1 Introduction

One of the primary causes of food loss is bacterial food spoilage. Biofilm build-up on raw ingredients or processing surfaces may contribute to the contamination of food items [1]. Biofilms are the consequence of the adhesion, growth and multicellular development of bacteria on a surface immersed in an extracellular matrix [2]. Biofilm formation can have negative effects on food processing and cause problems at food factories including contamination of food products [3], induction of corrosion in equipment, and reduction of the efficiency of heat transmission in specific processes and of production runs due to the need for cleaning equipment [4].

Biofilms can form under either static or dynamic settings, such as on surfaces, in containers, and pipelines. It is known that fluid flow significantly influence biofilm formation by affecting microbial surface adherence and colonization, as well as the composition of the biofilms [[5], [6], [7]]. Fluid flow can also influence adhesion, nutrient supply, and possibly the efficiency of chemical signalling molecules responsible for biofilm formation via quorum-sensing [8]. The flow regime controls the rate of delivery of macromolecules and microorganisms to surfaces. It also determines the shear stress at the solid-fluid interface important for initial adhesion of cells to the surface [9]. The surrounding fluid serves as both a source of nutrients and a means of removing cell waste [10]. Different stress mechanisms may alter biofilm development and consequently the physiological condition of biofilm cells. For example, under flow situations, higher oxidative stress could be increased due to constant exposure to oxygenated media.

Biofilm formation under static conditions is frequently employed for laboratory research using microtiter multi-well plates without and with additional placement of (submerged) coupons composed of different materials. This permits conventional analysis of different factors influencing biofilm formation [11]. On the other hand, flow conditions can be attained by growing the biofilm in a flow cell with a constant nutrient supply. Several methods, including rotating disk reactors, drip flow and microfluidics reactors, have been used to investigate biofilm formation under flow [[12], [13], [14], [15]].

Despite the importance of gram-positive bacteria in various food processing, environmental and clinical settings, only limited studies have evaluated the impact of flow on the formation and growth of biofilms of gram-positive bacteria. Studies on *Listeria monocytogenes, Bacillus subtilis*, and *Staphylococcus* species, revealed the importance of applying flow, ambient temperature and medium composition on biofilm formation and matrix structure [12,[16], [17], [18], [19]].

*Lactiplantibacillus plantarum* (previously, *Lactobacillus plantarum*) is a gram-positive, non-spore-forming, non-motile bacteria that can contaminate and cause food spoilage [20]. *Lactiplantibacillus* and *Lactobacillus* spp. Belong to the family of Lactic acid bacteria (LAB). LAB has been used for centuries as a starter culture in diverse fermented food products [21]. LAB can also be a source of contamination and can lead to food decline in a range of products including sliced meat, beer, salad dressings and ketchup (Xu et al., 2020). The majority of studies on the formation of LAB biofilms have been done under static conditions [19,20,[22], [23], [24]]. Previous studies on static biofilm formation of *L. plantarum* WCFS1 and six *L. plantarum* food isolates (FBR1-FBR6), showed that biofilm formation is boosted by added manganese and glucose (Fernández Ramírez et al., 2015). Exposure of the biofilms formed by the tested isolates to DNAse and proteinase K, showed that eDNA and/or proteinaceous material contribute to the extracellular polymeric substances (EPS) in these biofilms [25]. The limited number of studies that have been conducted on LAB biofilm formation under flowing conditions have not addressed the effects of flow on biofilm formation or the number of viable biofilm cells before and after exposure to disinfectants [20,21,26]. In a previous study, we found that the amount of crystal violet (CV)-stained biofilm material produced by the *L. plantarum* strain CIP104448 increased under flow, whereas the biofilm formation by the *L. plantarum* WCFS1 strain was not significantly affected under flow conditions, while for both strains an increase in biofilm cell counts (CFUs/well) was observed. Furthermore, DNase I and Proteinase K treatments reduced mature biofilms in WCFS1 under static conditions, while only DNase I had an effect on biofilms formed under flow conditions. Proteinase K decreased CV staining in both static and flow conditions for CIP104448, indicating distinct roles of extracellular DNA and proteinaceous matrix in biofilm formation for the studied strains [27].

In food industry several strategies are implemented to prevent biofilm formation on surfaces and (re)contamination of foods by spoilage and pathogenic bacteria, which is essential for production of high-quality, safe foods with a longer shelf-life. These strategies include hygienic design and the use of cleaning and disinfection procedures that involve compounds like hydrogen peroxide, peracetic acid, quaternary ammonium and chlorine-based compounds [[28], [29], [30]]. Therefore resistance to disinfectants is a very important issue not only in food production but also for medical applications. Cell history and the physiological state may affect the resistance to disinfectants. Previous studies showed that mechanisms underlying the resistance of biofilm cells to disinfectants can be diverse including protection offered by the EPS in the biofilm matrix via degradation, binding and/or restriction of diffusion of antimicrobials [31,32]. In addition, activation of nutrient-starvation and general stress defense, and generation of so-called non-growing persister cells may render bacterial biofilm cells resistant to antimicrobials [28,29,[33], [34], [35]].

The objective of this study was to examine how flow affects *L. plantarum* biofilm development and disinfectant resistance in various bacterial strains. We used a custom-designed setup that was introduced in our recent study (Rashtchi et al., 2022) and quantified biofilm formation of six *L. plantarum* food spoilage strains and a model strain WCFS1 under flow and static conditions. In addition, we investigated, the responses of planktonic cells and in situ and dispersed static and flow biofilm cells to peracetic acid (PAA), an oxidative agent, and benzalkonium chloride (BKC), a membrane damaging quaternary ammonium compound. The results obtained on the impact of static and flow conditions on biofilm formation and disinfectant resistance are discussed including the need for designing strategies to prevent biofilm-associated contamination and spoilage of foods.

## 2 Material and methods

### 2.1 Bacterial strains

We used *L. plantarum* WCFS1 as a reference strain (originally isolated from human saliva), alongside six *L. plantarum* strains associated with food spoilage. These spoilage isolates, named as FBR1 to FBR6, were sourced from various food products: FBR1, FBR2, FBR3 and FBR5 from salad dressing, FBR4 from cheese infused with garlic, and FBR6 from ketchup [25,36]. The strains were inoculated to De Man-Rogosa-Sharpe (MRS) broth from −80 °C stock and incubated for 18 h at 30 °C and used as an overnight culture for inoculation. Brain heart infusion (BHI) (Becton, Dickinson, France) supplemented with 0.005 % manganese sulphate and 2 % glucose (BHIMnG) was employed as a medium [25].

### 2.2 Flow system design

A custom-designed setup that was introduced in our recent study [27] was employed to investigate biofilm formation. The setup consisted of 48-well plates with a 3D-printed lid holding the inlet-outlet pipes. Each well's inlet and outlet were connected to two syringes (fixed in syringe pumps) via plastic tubes so that a constant flow rate of culture media could be injected into them and removed. In addition to the static condition (no flow), a flow rate of 3.2 ml/h was imposed in the dynamic experiments. Given the medium volume per well (0.8 ml), the chosen flow rate led to four-volume changes per hour (every 15 min), which is well above the previously determined doubling times of *L. plantarum* strains WCFS1 and CIP of 136 min for both. (Rashtchi et al., 2022). The experiment duration was 24 h.

### 2.3 Biofilm analysis

#### 2.3.1 Biofilm formation and crystal violet assay

The microtiter plates were filled with 0.8 ml BHIMnG. Wells of microtiter plate were inoculated with 12 μl of overnight grown MRS broth (OD_600_ = 5). The plate was placed for 24 h in an incubator (New Brunswick™ Innova) at 30 °C. Biofilm formation was determined by crystal violet staining [25]. In this assay, a pipette is used to gently remove the liquid content of each well. To remove unattached cells the wells with formed biofilm were washed three times with 900 μl phosphate-buffered saline of pH 7.4 (PBS, KCl 0.2 g/l; NaCl 8 g/l; KH2PO4 0.24 g/l; Na2HPO4 1.44 g/l; (Merck)). The resulting biofilm was stained with 800 μl of 0.1 (w/v) of crystal violet (Merck) for 30 min at room temperature. Stained wells were washed three times with 900 μl PBS using a micropipette to remove excess crystal violet. Retained crystal violet was dissolved in 800 μl 70 % ethanol for 45 min; in the next step, 200 μl of dissolved crystal violet was transferred to a 96-wells microplate. Finally, the absorption at 595 nm wavelength was measured using a microplate reader (SpectraMax, Molecular Device).

#### 2.3.2 Quantification of colony-forming units

The plate counting method was employed to measure the number of culturable cells in the biofilm after 24 h (biofilm cells). The liquid content of the incubated plate was removed via pipetting. Then the biofilm was washed three times with PBS (900 μl) to remove the unattached cells and resuspended in 1 ml PBS by scraping. Peptone Physiological Salt Solution (PPS) was used for serial dilution of cells. Finally, they plated on MRS agar. CFUs were determined after 48 h incubation at 30 °C.

### 2.4 Live/dead cells fluorescence imaging

Dead/live cell tests were performed based on nucleic acid and membrane staining [37]. Both, membrane-permeant (SYTO9) and a non-membrane permeant (propidium iodide) DNA-binding fluorophores were used for staining the biofilm. SYTO9 can pass the cell membrane and emits green fluorescence following binding to the DNA of both live and dead bacterial cells. On the other hand, propidium iodide (PI) cannot pass the cell membrane. Therefore, it only emits red fluorescence after binding to DNA of dead cells with ruptured membranes. For preparing a sample for microscopic visualization, first, the culture medium was removed carefully using a micropipette. Then the wells were washed with 900 μl PBS, three times. The remaining biofilm in the wells was resuspended by scraping in 1 ml of PBS and spun down by using a centrifuge (8000 g, 5 min). Cell biofilm pellets were resuspended in 200 ml PBS and the cell suspension was stained with 1 μl of SYTO9 (0.0334 mM) and 1 μl PI (0.2 mM). The excitation/emission maxima are ∼480/500 nm and ∼490/635 nm for SYTO9 and PI, respectively. An axioskope epifluorescence fluorescence microscope equipped with a 50 W mercury lamp was used for microscopy. A Plan-Neofluar 100× objective lens, a filter set with emission wavelength >500 nm and excitation wavelength 450–490 nm and Carl Zeiss CCD (Carl Zeiss, Germany) were employed for recording the fluorescent images.

### 2.5 Disinfection treatments

The 48-well microtiter plates containing 0.8 ml BHI-Mn-G were inoculated with 12 μl Overnight (18 h) grown cultures at 30 °C and incubated at 30 °C for 24 h. Cells originated from resuspended biofilm, cells within the biofilm, and Planktonic cells were exposed to 1 ml of 100 μg/ml benzalkonium chloride and 20 μg/ml peracetic acid for 10 min under static conditions at room temperature. For disinfection treatment of planktonic cells, planktonic cultures were grown statically on 48-well polystyrene microtiter plates for 24 h at 30 °C. Then the supernatant in a well was centrifuged (5 min at 8000 g). After three times washing with PBS, the final pellet was resuspended for 10 min in 1 ml of disinfectant agents. The cells were serial diluted in PPS and plated on MRS at 30 °C for 48 h. For disinfection treatment of the cells within the biofilm, the formed biofilms under both static and flow conditions were washed twice with PBS and exposed to disinfectant agents for 10 min. After exposure, the biofilms were washed once with PBS and resuspended in 1 ml PBS, again the cells were serially diluted in PPS and plated on MRS at 30 °C for 48 h. Disinfection treatment of resuspended biofilm cells was as follows: the formed biofilms under both static and flow conditions were washed three times with PBS, then the biofilm was resuspended in 1 ml of disinfectant agents and incubated at room temperature for 10 min. The cells were serial diluted in PPS and plated on MRS at 30 °C for 48 h [38].

### 2.6 Data analysis

To determine the significance of flow on the total biofilm, viable and dead cells and also the effect of disinfectant agents on planktonic and biofilm cells, the one-way analysis of variance (ANOVA) followed by Duncan was applied in SPSS. P value smaller than 0.05, was considered significantly different. All the experiments of this study were performed in three independent biological replicates and each biological replicate includes three technical replicates.

## 3 Results

### 3.1 CV staining of *L. plantarum* WCFS1 and FBR 1–6 biofilms under static and flow conditions

**Fig. 1:**
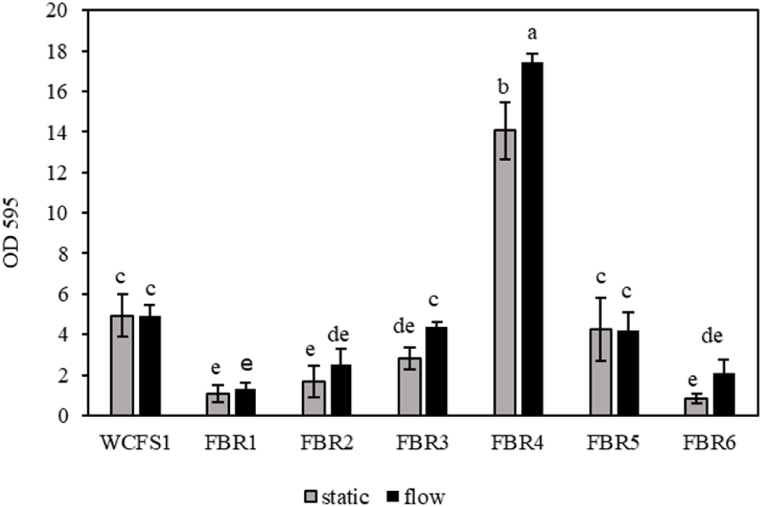
Different stains of *L. plantarum* (WCFS1 and FBR1-FBR6) formed biofilm under static and dynamic conditions. Grey and black bars represent CV staining (OD595 values) of biofilms formed after 24 h in static and flow conditions (flow rate of 3.2 ml/h), respectively. Different letters indicate the results of the Duncan analysis with a significant difference at p smaller than 0.05. The same letters indicate that there is no significant difference. From highest to lowest value, the range is from a to e.

The amount of formed biofilm by *L. plantarum* WCFS1 and FBR1-FBR6 strains under static conditions and under flow (3.2 ml/h), was measured after 24 h using CV staining (Fig. 1).

The highest CV-stained static biofilm formation was observed for FBR4, followed by WCFS1 and FBR5, and then by FBR3, FBR2, FBR1 and FBR6, respectively. Under flow conditions, strains FBR3 and FBR4 formed significantly higher amounts of CV-stained biofilms, with the latter strain again producing the highest amount of biofilm, while the other strains showed no significant changes biofilm formation. In static conditions, all tested strains formed clear biofilms at the bottom of the well, with some additional CV-stained rings at the wall of the well at the air-water interface, especially for FBR2. Notably, only FBR4 showed additional CV-stained biofilm formation on the vertical wall of the well.

### 3.2 Quantification of culturable biofilm cells under different conditions

**Fig. 2:**
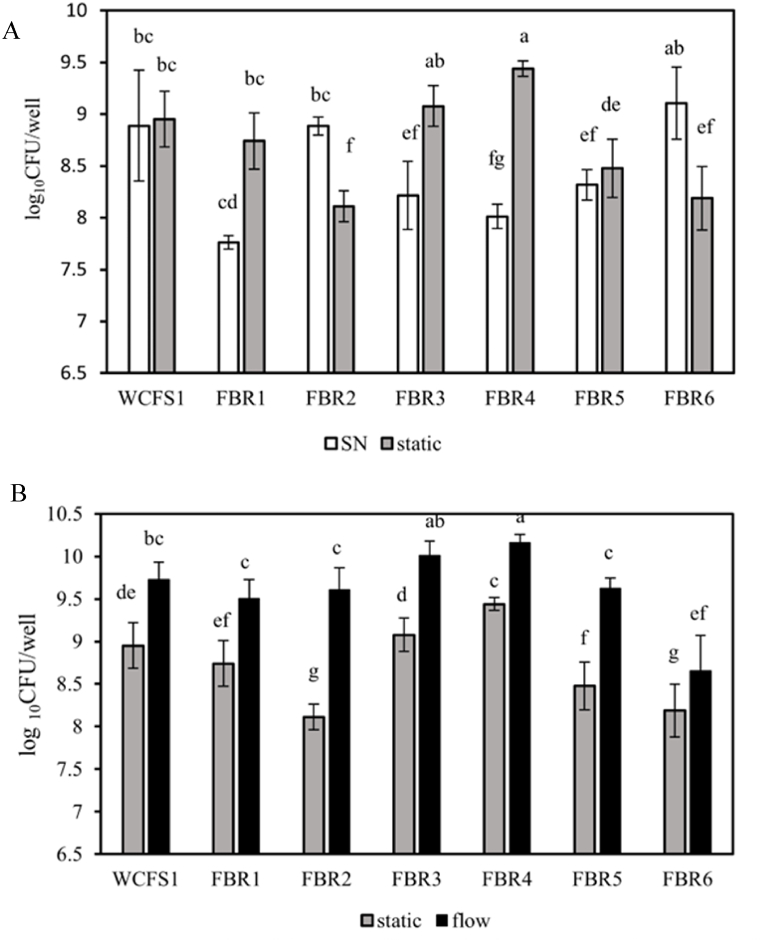
The number of culturable cells in supernatant and biofilms formed by *L. plantarum* WCFS1 and FBR1-FBR6 after 24 h under static and 3.2 ml/h flow conditions. (A) Comparison of cell counts (CFU/ml) in supernatants (white bars) and static biofilms (grey bars). (B) Comparison of cell counts (CFU/ml) in biofilms under static (grey bars) and flow conditions (black bars). Different letters indicate the results of the Duncan analysis with a significant difference at p smaller than 0.05. The same letters indicate that there is no significant difference: a to-go signifies from highest to lowest value.

We next determined the number of culturable cells in the biofilms formed by *L. plantarum* WCFS1 and FBR1-FBR6 under static and flow conditions after 24 h (Fig. 2). In static conditions (Fig. 2A) culturable cell numbers in the supernatant ranged from appr 8 log_10_ CFU/well for strains FBR1, FBR3, FBR4 and FBR5, to approximately 9 log_10_ CFU/well for strains WCFS1, FBR2 and FBR6. Notably, static biofilm cell counts compared to corresponding supernatant cell counts were similar for WCFS1 and FBR5 and lower for FBR2 and FBR6.

Additionally, the biofilm cell counts for FBR1, FBR3, and FBR4 were higher than the corresponding supernatant cell counts, with the latter strain (FBR4) reaching the highest static biofilm cell counts.

Quantification of culturable cells in biofilms formed under static and flow conditions showed significantly higher cell numbers in the latter condition for all tested strains, with the highest numbers for FBR3 and FBR4 (about 10 log_10_ CFU/well), followed by strains WCFS1, FBR1, FBR2 and FBR5 (about 9.5 log_10_ CFU/well), and the lowest number for FBR6 (8.5 log_10_ CFU/well) (Fig. 2B). The increase of flow biofilm cell counts of FBR3 and FBR4 matches the significant increase of the corresponding CV values presented in Fig. 1.

### 3.3 Correlation between cell counts and CV staining results

**Fig. 3:**
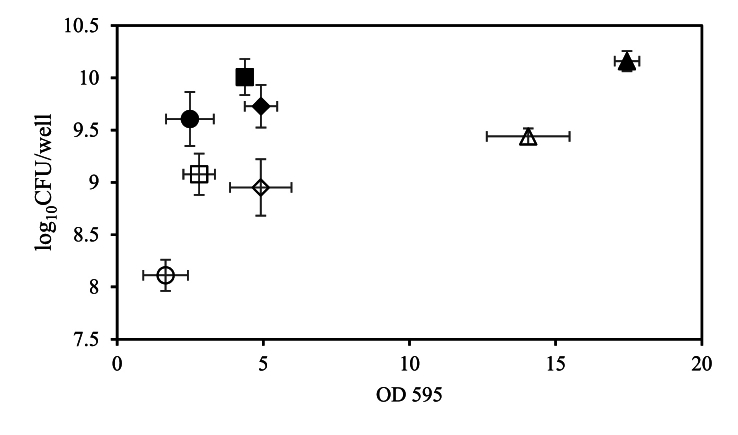
The biofilm cell count data versus biofilm CV staining is shown for each *L. plantarum* strain under static and flow conditions. Different symbols represent different experiments: strain WCFS1 (diamonds), FBR2 (circles), strain FBR3 (squares), and FBR4 (triangles). Open symbols indicate static conditions, while closed symbols represent flow conditions.

After statistical analyses of the results presented in Fig. 1, Fig. 2 we found scatter data in the plot of log10 CFUs/well versus the OD 595 nm as shown in Fig. 3. The scattered data indicates that there was not a strong correlation between the cell counts and CV staining results.

### 3.4 Fluorescence images of biofilms

Next, dispersed biofilm samples formed under static and flow conditions were stained with membrane-permeant and non-permeant DNA-binding fluorescent probes Syto9 and propidium iodide (PI), to indicate next to extracellular DNA (eDNA), live and dead cells with intact and damaged membranes, respectively.

**Fig. 4:**
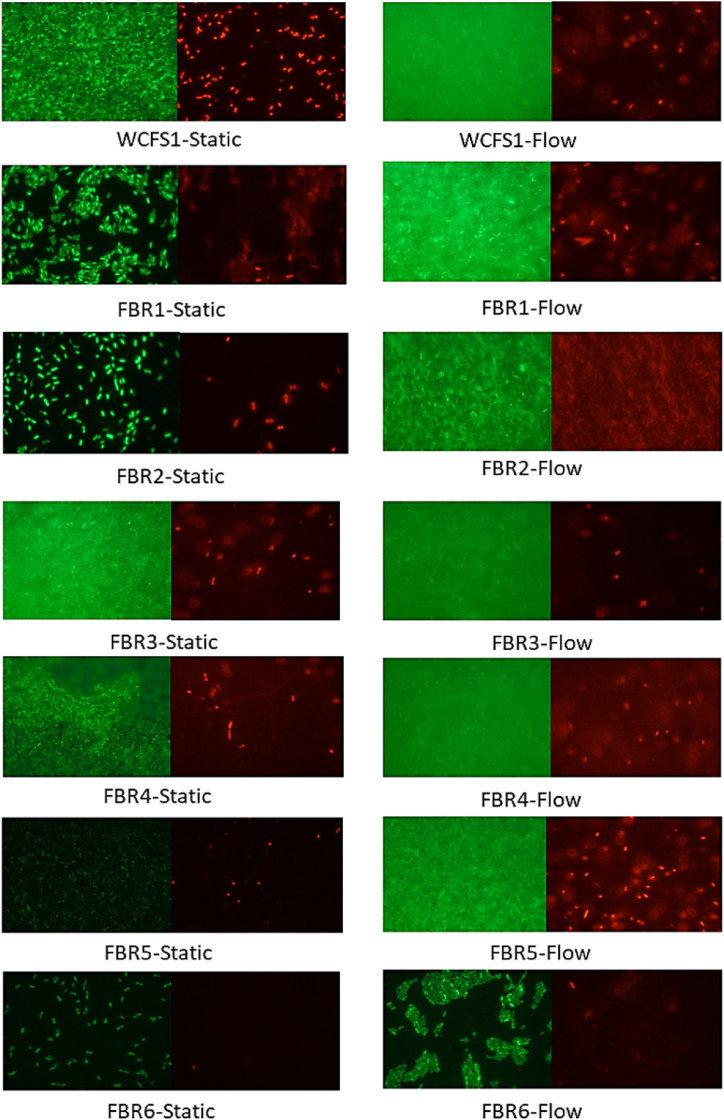
Fluorescence images of samples from biofilms formed *by L. plantarum* WCFS1 and FBR1-FBR6 under static and flow (3.2 ml/h) conditions. Cells were stained with LIVE/DEAD BacLight viability kit: Syto9 (left) and propidium iodide (right). Green and red colors represent alive and dead cells, respectively.

Fluorescence microscopy images in Fig. 4 show that for all tested strains the density of live cells increased under flow conditions. This is in agreement with the results of biofilm cell counts presented in Fig. 2. The low number of live cells in FBR2, FBR5 and FBR6 biofilm samples taken under static conditions is apparent in the fluorescence images (Fig. 4). Notably, in FBR3 and WCFS1 biofilms, the apparent ratio of dead cells to living cells is lower in biofilm samples from flow conditions than in static conditions. It is conceivable that the higher amount of CV-stained biofilms in FBR4 compared to FBR3 and WCFS1 is partly related to an increased number of dead cells and/or eDNA.

### 3.5 Effect of disinfectants on planktonic and biofilm cells

Planktonic cells (SN), cells (in situ) within the biofilm and resuspended biofilm cells were exposed to 1 ml of 100 μg/ml benzalkonium chloride (BKC) and 20 μg/ml peracetic acid for 10 min at room temperatures (Fig. 4). We used strains WCFS1, FBR2, FBR3, and FBR4 to study the effect of disinfection on biofilms formed in static conditions and under flow after 24 h. These strains were selected because FBR3 and FBR 4 formed higher amounts of CV-stained biofilm under flow conditions compared to the static condition, FBR2 showed the largest increase in flow biofilm cell counts compared to static biofilm cell counts (>1.5 log_10_ CFU/well), and WCFS1 is used as a reference strain.

**Fig. 5:**
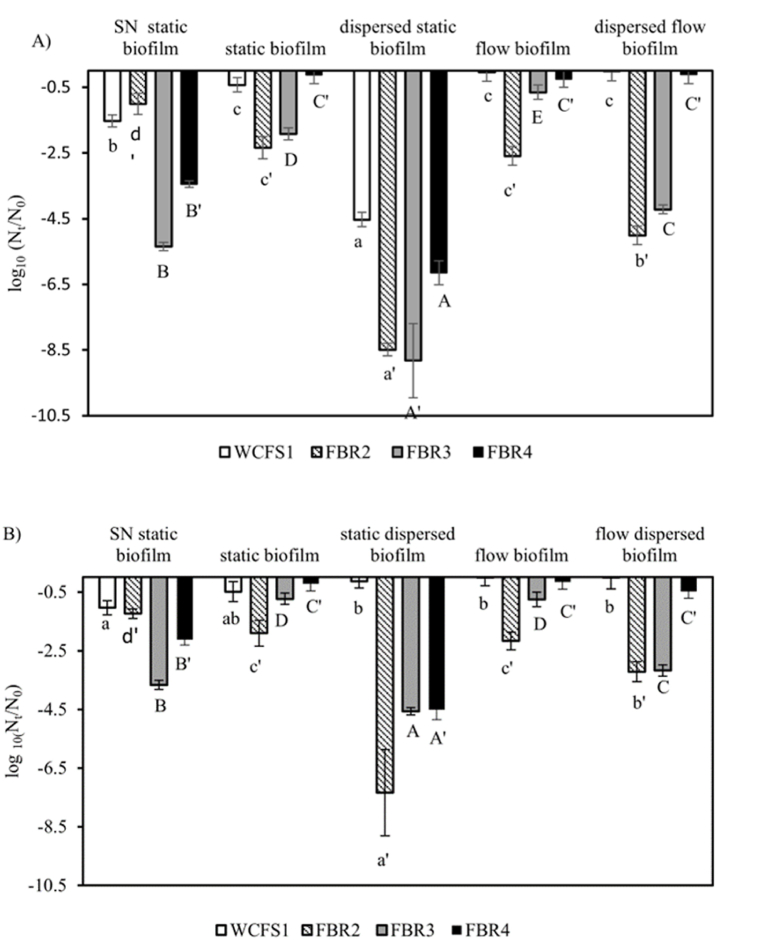
Inactivation of planktonic cells, biofilm cells, and resuspended biofilm cells of selected strains of *L.plantarum* after disinfection treatment. N_0_ is the number of culturable cells before treatment and N_t_ is the number of cells after disinfection treatment. The disinfectant agents used were (A) 100 μg/ml benzalkonium chloride and (B) 20 μg/ml peracetic acid. The strains tested were WCFS1(white bars), FBR2 (patterned bars), FBR3 (grey bars), and FBR4 (black bars). SN indicates cell counts in the supernatant of static biofilm cultures. Different letters indicate a significant difference at P smaller than 0.05 based on the results of the Duncan analysis. The same letters indicate that there is no significant difference. From highest to lowest value, the range is from a to c.

Results presented in Fig. 5A show that treatment with BKC led to higher inactivation of FBR3 and FBR4 supernatant cells compared to WCFS1 and FBR2. Static and dynamic biofilm cells of WCFS1, FBR3, and FBR4 were found to be more resistant than their supernatant counterparts, whereas FBR2 biofilm cells were more sensitive than the corresponding supernatant cells. Notably, there was limited and even minimal in situ inactivation of static biofilm cells of FBR3, WCFS1, and FBR4, respectively, while dispersed static biofilm cells of all four tested strains were highly sensitive to BKC. In contrast, dispersed flow biofilm cells of WCFS1 and FBR4 exhibited no and limited inactivation, respectively, similar to that of the corresponding in situ flow biofilm cells. On the other hand, dispersed flow biofilm cells of FBR2 and FBR3 were more sensitive than their in-situ counterparts.

**Fig. 6:**
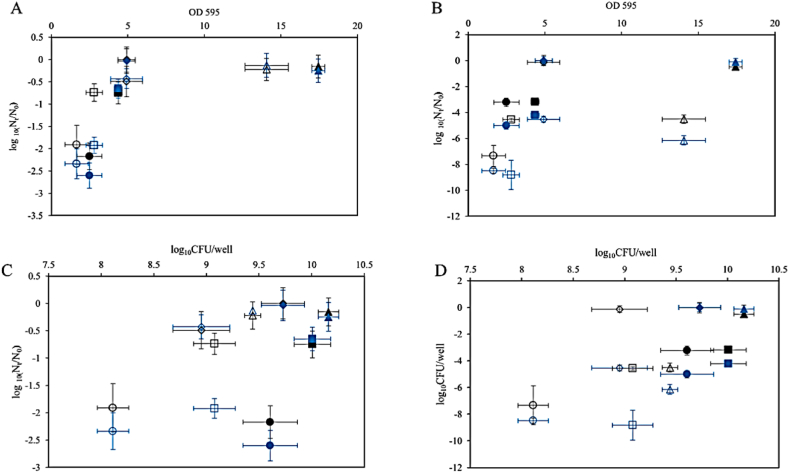
Overview of selected correlations. A. Correlation biofilm CV staining and disinfectant-induced biofilm cell inactivation, B. Correlation biofilm CV staining and disinfectant-induced resuspended biofilm cell inactivation, C. Correlation biofilm cell counts and disinfectant-induced biofilm cell inactivation, and D. Correlation biofilm cell counts and disinfectant-induced resuspended biofilm cell inactivation. Symbols used represent strain WCFS1 (diamonds), FBR2 (circles), strain FBR3 (squares), and FBR4 (triangles), with open and closed symbols representing data obtained in static conditions and flow conditions and following exposure to PAA (black) and BKC (blue), respectively.

Similar results were obtained following exposure to PAA (Fig. 5B), with static and dynamic biofilm cells of FBR2 showing higher sensitivity to PAA than the supernatant cells. Notably, while WCFS1 dispersed static biofilm cells showed limited inactivation upon exposure to PAA, dispersed FBR2, FBR3, and FBR4 cells exhibited high sensitivity to PAA (>4.5 log_10_ inactivation). Dispersed flow biofilm cells of WCFS1 and FBR4 showed again very limited inactivation by PAA, whereas dispersed flow biofilm cells of FBR2 and FBR3 showed higher sensitivity than in situ biofilm cells (about 3.5 log_10_ versus 1–2 log_10_ inactivation), but lower sensitivity than dispersed static biofilm cells (about 7 and 5 log_10_ inactivation). Combining all results obtained following exposure to PAA and BKC, correlation analysis in Fig. 6A–D indicates there is no correlation between the biofilm cell counts and the resistance of (resuspended) biofilm cells. However, biofilm CV staining and disinfectant-induced resuspended biofilm cell inactivation show a positive correlation (Fig. 6 B) under both static and flow conditions, which points to a potential protective effect of biofilm matrix and/or components against the tested disinfectants. Overall, except for FBR2, static and flow biofilm growth modes provided strong protection of WCFS1, FBR3 and FBR4 against disinfection agents, while dispersed static biofilm cells were very sensitive to BKC and, with the exception of WCFS1, also to PAA. The high resistance against BKC and PAA of dispersed flow biofilm cells of WCFS1 and FBR4 is conceivably linked to stress defense activation due to in situ microenvironments encountered in biofilms formed under flow conditions.

## 4 Discussion

In our recent study we used a custom-designed fluidic device to perform a comparative analysis of biofilm formation by *Lactiplantibacillus plantarum* strains WCFS1 and CIP104448 in static conditions and under flow showing significant differences between the two tested strains after 24 h incubation [27]. In the current study, we used this approach to quantify biofilm formation of six *L. plantarum* food spoilage-isolates, FBR1-FBR6, using the model strain *L. plantarum* WCFS1 as a benchmark, and determine the impact of peracetic acid (PAA), an oxidative agent [39], and benzalkonium chloride (BKC), a membrane targeting agent (Jaramillo et al., 2012), on planktonic cells and on in situ and resuspended static and dynamic flow biofilm cells. The obtained results showed a striking diversity in biofilm formation capacity and disinfectant resistance between strains and cell types, pointing to the relevance of studying both static and dynamic flow biofilm characteristics.

In the static conditions, *L. plantarum* strain FBR4 produced the highest amount of CV-stained biofilm material, followed by WCFS1 and FBR5. Conversely, the lowest CV-stained biofilm formation was observed in FBR3, FBR2, FBR1, and FBR6, respectively. These findings are in line with previous results reported by Fernández Ramírez and coworkers [25]. The overall biofilm's development, as measured by CV staining, may have resulted from an increase in the number of cells (dead and alive) and/or matrix components. The highest number of static biofilm cells was observed for FBR4, followed by FBR3 and WCFS1, and the lowest numbers for FBR1, FBR5, FBR2 and FBR6, respectively. The difference in the trend of culturable cells and the amount of formed biofilm (CV staining result) could be due to the presence of CV stainable matrix components such as eDNA, proteins and proteinaceous material [25,27]. Previous findings of Fernández Ramírez et al. (2015) indicated that prolonging the incubation time up to 48 and 72 h resulted in a further increase in CV staining and a drop in the number of culturable cells [25].

Biofilm formation of WCFS1 and FBR1-FBR6 under flow conditions revealed, only significantly higher CV staining for strains FBR3 and FBR4, indicating a substantial increase in biofilm production compared to that in static conditions. In our previous study, we also observed diversity in performance of two tested with *L. plantarum* strains, WCFS1 and CIP 104448, with only the latter strain showing increased biofilm formation under flow (3.2 ml/h) [27]. Flow can have different effects on biofilm formation, for example via the so-called shear-trapping effect [14] which may translocate bacteria to high-shear regions along the walls and enhance the possibility of cell attachment to the surface, i.e., the initial stage in biofilm formation. Besides increasing the efficacy of attachment, shear stress may induce the production of extracellular polymeric substances (EPS), which supports biofilm growth and reduces dispersion of mature biofilms under flow conditions [40]. In the current study, the increase of CV-stained FBR3 and FBR4 biofilms under flow conditions is conceivably linked to the increase in the number of alive or/and dead cells, and the presence of eDNA and proteinaceous material as previously reported for static *L. plantarum* biofilms [25,27]. Application of flow significantly increased the number of culturable cells in the biofilm in all strains which is conceivably inked to the continuous supply of fresh nutrient-rich medium in these conditions, in line with previous studies on bacterial biofilms produced under flow [27,41,42]. Additional information supplied by fluorescence microscopy also showed a higher density of Syto9-stained viable biofilm cells under flow conditions. Under flow conditions, biofilm cell counts of FBR3 and FBR4 were comparable, however, CV staining of FBR4 biofilms was four times higher than that of FBR3, which correlates with a higher density of PI-stained dead cells in FBR4 biofilms. Consequently, the higher CV staining of FBR4 biofilms under flow is conceivably linked lysis of cells and the release of proteinaceous material and/or (e)DNA.

In the worldwide food business, disinfectants are frequently used to inactivate microorganisms on surfaces to prevent microbiological contamination of raw materials and food products (Yuan et al., 2021). In the current project, we aimed to investigate the impact of flow on the resistance of biofilm cells to disinfection. To assess this, we utilized peracetic acid (PAA) as an oxidative agent and benzalkonium chloride (BKC) as a quaternary ammonium compound to disinfect both planktonic cells and in situ and resuspended cells from biofilms formed under static and flow conditions. PAA is a disinfectant with significant oxidizing properties due to the production of hydroxyl radicals that cause damage to the cellular proteins and DNA leading to cell inactivation [43]. BKC's mechanism of action against bacterial cells is likely to include a general disruption of lipid bilayer membranes, resulting in the leakage of cytoplasmic materials into the environment [44]. We found that the cells within biofilms formed by WCFS1, FBR3, and FBR4 under both static and flow conditions exhibited higher resistance to disinfection agents compared to planktonic cells, while biofilm growth modes did not contribute to the survival of FBR2. It is conceivable that the observed higher resistance of in situ static biofilm cells compared to dispersed biofilm cells from all four tested strains to BKC and of FBR2, FBR3, and FBR4 to PAA, is due to binding of the disinfectants to matrix components and/or their limited access to the biofilm cells.

Furthermore, dispersed flow biofilm cells of FBR2 and FBR3 were also more sensitive to disinfecting treatment than in situ biofilm cells. These findings indicate that the effectiveness of BKC and PAA against the biofilms formed under flow conditions was reduced, conceivably due to factors such as cell density, aggregation of cells, and the presence of matrix components. In addition, cell physiology was conceivably affected by differences in situ microenvironments in biofilms [45]. In the flow biofilm settings in the current study, biofilm cells were exposed to shear stress and conceivably to higher levels of nutrients and lower levels of metabolites and other waste products, which in turn could induce strain-specific changes in the physiological state linked to reduced (FBR2 and FBR3) and enhanced (FBR4 and WCFS1) resilience.

## 5 Conclusion and outlook

Our findings reveal that certain bacterial strains form more biofilm under flow conditions than under static conditions, and these biofilms contain more culturable cells than their static counterparts. This implies an increased risk of contamination following the dispersal of biofilm material. Furthermore, we observed that biofilm cells of certain strains displayed greater resistance to disinfection compared to their planktonic counterparts, especially in the case of resuspended flow biofilm cells. Further studies using proteomics analysis could support determining the relationship between the disinfection resistance of dispersed biofilm cells and the activation of specific metabolic pathways and stress-defense proteins. Our findings highlight the necessity for further exploration of the potential food quality issues linked to biofilm formation of non-motile *L. plantarum* under flow conditions. The methodology and results of our study can serve as a foundation for future investigations in this field including the testing of (combinations of) novel (natural) antimicrobials and disinfectants that support the translation to industry and the development of customized sustainable disinfection strategies to effectively remove biofilms in food production settings.

## Data availability statement

All raw data reported in this work is available and can be provided upon request.

## CRediT authorship contribution statement

**P. Rashtchi:** Writing – original draft, Methodology, Investigation, Formal analysis, Data curation. **E. van der Linden:** Writing – review & editing, Supervision, Conceptualization. **M. Habibi:** Writing – review & editing, Writing – original draft, Supervision, Project administration, Methodology, Conceptualization. **T. Abee:** Writing – review & editing, Supervision, Methodology, Investigation, Conceptualization.

## Declaration of competing interest

The authors declare the following financial interests/personal relationships which may be considered as potential competing interests: Mehdi Habibi reports financial support and article publishing charges were provided by 10.13039/501100004890Wageningen University. Mehdi Habibi is the editor-in-chief of the Journal of Applied Rheology. If there are other authors, they declare that they have no known competing financial interests or personal relationships that could have appeared to influence the work reported in this paper.
